# Genetic Analysis and Evolutionary Changes of the Torque teno sus Virus

**DOI:** 10.3390/ijms20122881

**Published:** 2019-06-13

**Authors:** Gairu Li, Wenyan Zhang, Ruyi Wang, Gang Xing, Shilei Wang, Xiang Ji, Ningning Wang, Shuo Su, Jiyong Zhou

**Affiliations:** 1 MOE International Joint Collaborative Research Laboratory for Animal Health & Food Safety, Jiangsu Engineering Laboratory of Animal Immunology, Institute of Immunology, College of Veterinary Medicine, Nanjing Agricultural University, Nanjing 210000, China; ligru2018@163.com (G.L.); Wenyan__Zhang@163.com (W.Z.); 2017107069@njau.edu.cn (R.W.); vet_doc@126.com (S.W.); 17416104@njau.edu.cn (N.W.); 2 Key Laboratory of Animal Virology of Ministry of Agriculture, Zhejiang University, Hangzhou 310027, China; gxing@zju.edu.cn; 3 Department of Biomathematics, University of California, Los Angeles, CA 90095, USA; xji3@ucla.edu

**Keywords:** TTSuV1, TTSuVk2, *Sus scrofa*, *Sus scrofa domestica*, natural selection, adaptation

## Abstract

The torque teno sus virus (TTSuV) is an emerging virus threating the *Suidae* species of unclear pathogenicity, although it was previously reported as a worsening factor of other porcine diseases, in particular, porcine circovirus associated disease (PCVAD). Here, a comprehensive codon usage analysis of the open reading frame 1 (ORF1), which encodes the viral capsid protein, was undertaken for the first time to reveal its evolutionary history. We revealed independent phylogenetic processes for the two genera during TTSuV evolution, which was confirmed by principal component analysis (PCA). A low codon usage bias was observed in different genera and different species, with Kappatorquevirus a (TTSuVk2a) displaying the highest, which was mainly driven by mutation pressure and natural selection, especially natural selection. Overall, ATs were more abundant than GCs, along with more A-ended synonymous codons in relative synonymous codon usage (RSCU) analysis. To further confirm the role of natural selection and TTSuV adaptation to the *Suidae* species, codon adaptation index (CAI), relative codon deoptimization index (RCDI), and similarity index (SiD) analyses were performed, which showed different adaptations for different TTSuVs. Importantly, we identified a more dominant role of *Sus scrofa* in the evolution of *Iotatorquevirus* (TTSuV1), with the highest CAI values and lowest RCDI values compared to *Sus scrofa domestica*. However, in TTSuVk2, the roles of *Sus scrofa* and *Sus scrofa domestica* were the same, regarding codon usage, with similar CAI and RCDI values. Our study provides a new perspective of the evolution of TTSuV and valuable information to develop control measures against TTSuV.

## 1. Introduction

The torque teno virus (TTV) was first identified in *Homo sapiens* and then in numerous host species and, therefore, was considered to have a broad host range [1,2]. TTV infecting the *Suidae* species is named the torque teno sus virus (TTSuV). Until now, the pathogenicity of TTSuV was considered uncertain, with an unclear role as a negative factor for other diseases, especially porcine circovirus associated disease (PCVAD) [3]. Similar to porcine circovirus, the genome of TTSuV is a single-stranded, negative-sense circular DNA of 2.8 kb [4]. TTSuV belongs to two different genera, *Iotatorquevirus* (TTSuV1) and *Kappatorquevirus* (TTSuVk2) of the *Anelloviridae.* The TTSuV1a and TTSuV1b species belong to the *Iotatorquevirus* genera, while TTSuVk2a and TTSuVk2b belong to the *Kappatorquevirus* genera, based on nucleotide divergence [5].

The TTSuV genome includes a conserved untranslated region (UTR) with a short GC-rich region, a major open reading frame (ORF1) encoding the viral capsid protein, and two ORFs encoding non-structural proteins: ORF2 is responsible for viral replication and suppression of the NF-kB pathway and ORF3 is of unknown function [6,7]. Previous TTSuV evolutionary analysis was based on the genomic fragment spanning the UTR, the complete ORF2, and partial ORF1 [8,9]. Evolutionary analysis on DNA viruses has been revealed that the high nucleotide substitution rate of TTSuV is consistent with the porcine circovirus type 3 (PCV3) [10], as well as other ssDNA and RNA viruses suspected to be driven by natural selection and drift [8,11], and higher than the pseudorabies virus (PRV) [12].

A direct way to display the evolutionary changes of viruses is the analysis of the codon usage pattern [13]. Each amino acid can be translated at least by one triplet codon, indicative of the redundancy of the genetic code. Codons encoding the same amino acid are referred to as “synonymous” codons. Importantly, the frequency of use of synonymous codons is biased, both in prokaryotes and eukaryotes; a phenomenon known as “codon usage bias” [14]. Some of the factors determining the codon usage pattern include mutation pressure, natural selection, selective transcription, external environment, etc. [15]. TTSuV, as any other virus, depends on the host for survival and transmission; therefore, the TTSuV codon usage pattern could influence virus infection, adaptation, and escape from host immune responses.

Epidemiological studies discovered that TTSuV is ubiquitous in domestic pigs [16] and wild boars [17]. However, most studies focused on domestic pigs. Whether different TTSuV genera experienced distinct evolutionary changes, and if these changes are influenced by the host, remains unknown. In this study, we investigated TTSuV evolution based on the ORF1 gene, encoding for the viral capsid protein and occupying most of the genome. In particular, we performed a comprehensive codon usage analysis that could aid the understanding of virus-host inter-adaption and thus inform surveillance and prevention strategies.

## 2. Results

### 2.1. Recombination and Phylogeny

According to the International Committee on Taxonomy of Viruses (ICTV) (https://talk.ictvonline.org/taxonomy/), TTSuV1 and TTSuV2 belong to different genera, *Iotatorquevirus* and *Kappatorquevirus*, given their high divergence (>56%). Therefore, the two genera were analyzed independently. A total of 181 sequences published on the National Center for Biotechnology Information (NCBI) GenBank (https://www.ncbi.nlm.nih.gov/genbank/) were chosen for analysis.

Initial analysis revealed that one TTSuV1 sequence (HM170069) and 11 TTSuVk2a sequences (JF937657, JF937656, GU180046, GU376737, JX535332, KR054748, JF937659, KR054750, A872, A003, and A907) experienced recombination and were, therefore, excluded from further analysis.

**Figure 1:**
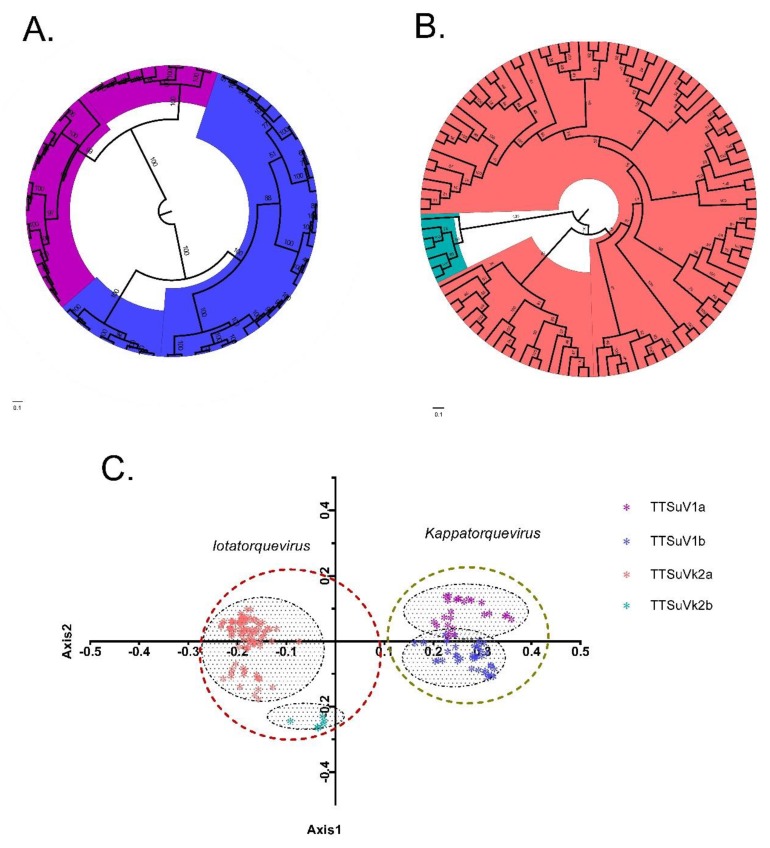
Maximum likelihood (ML) tree of the torque teno sus virus (TTsuV) *Iotatorquevirus* (TTSuV1) (**A**) and *Kappatorquevirus* (TTSuVk2) (**B**) reconstructed by RAxML (v8.2.10). Principal component analysis (PCA) in terms of codon usage pattern (**C**). TTSuV1a, TTSuV1b, TTSuVk2a, and TTSuVk2b are represented in purple, blue, pink, and green, respectively.

Phylogenetic analysis revealed that TTSuV1 and TTSuVk2 clustered into two groups: TTSuV1a and TTSuV1b, and TTSuVk2a and TTSuVk2b, respectively (Figure 1A,B). The combined nucleotide heterogeneity was >35% [5].

### 2.2. Principal Component Analysis

The first two axes accounted for the majority of the total variation (54.84%), therefore they were chosen for principal component analysis (PCA). As shown in Figure 1C, the *Iotatorquevirus* and *Kappatorquevirus* genera grouped separate from each other. In contrast, TTSuV1a and TTSuV1b clustered closer together than TTSuVk2a and TTSuVk2b, in agreement with the phylogenetic distribution.

### 2.3. Codon Usage Analysis

#### 2.3.1. Nucleotide and Codon Composition

**Figure 2:**
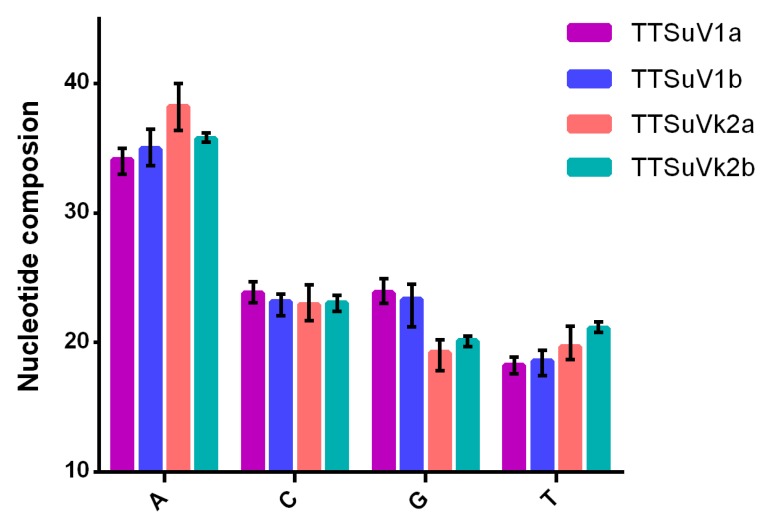
Nucleotide composition of TTSuV1a, TTSuV1b, TTSuVk2a, and TTSuVk2b. Nucleotides A, T, C, and G are represented by purple, blue, pink, and green, respectively.

Nucleotide A was the most abundant, regardless of species (Figure 2), followed by nucleotides G, C, and T in TTSuV1 and G, C, and T in TTSuVk2. In addition, ATs were more abundant than GCs. Furthermore, in terms of codon composition, A was the most abundant nucleotide, being more than 50% among all the codons at the third position regardless of genera (Supplementary Materials).

#### 2.3.2. RSCU in Different Species

Within the 18 most abundant synonymous codons, A-ended codons were the most frequent among the four synonymous codons, with an occurrence ten times in TTSuV1 and eight times in TTSuVk2. The frequencies of the other three synonymous codons were: five for C-ended, two for T-ended, and one for G-ended in TTSuV1, while six for T-ended and four for C-ended in TTSuVk2 (Table 1). In addition, eight (CTA, GTA, AGT, CCA, ACA, GCA, AGA, GGA) and nine (CTA, ATA, GTA, AGC, ACT, GCT, AAA, AGA, GGA) preferred codons had relative synonymous codon usage (RSCU) values of more than 1.6, indicating over-represented codons in TTSuV1 and TTSuVk2, respectively, and a higher frequency of A-ended synonymous codons. In addition, the RSCU relative to species was performed. We found that in TTSuV1, the 18 most abundant synonymous codons displayed no difference, whereas distinct RSCU patterns were observed in TTSuVk2a and TTSuVk2b. Moreover, to analyze the impact of the host on the RSCU pattern of TTSuV, the RSCU value of reference hosts, *Sus scrofa*, *Sus scrofa domestica,* and TTSuV, were compared. No complete coincidence nor antagonism existed among them. The ratio of coincidence/antagonism in TTSuV1 was 6/12 (both in TTSuV1a and TTSuV1b) compared to *Sus scrofa* and 11/7 compared to *Sus scrofa domestica* while it was 2/16 (except 4/14 in TTSuVk2a) in *Sus scrofa* and 5/13 (7/11 in TTSuVk2a and 6/12 in TTSuVk2b) in *Sus scrofa domestica* in TTSuV2 (Figure S1).

#### 2.3.3. TTSuV Codon Usage Bias

**Figure 3:**
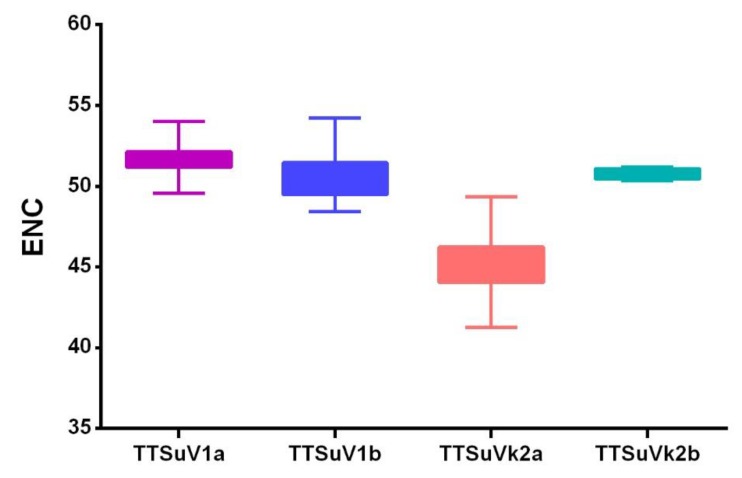
Effective number of codons (ENC) of TTSuV. TTSuV1a, TTSuV1b, TTSuVk2a, and TTSuVk2b are represented in purple, blue, pink, and green, respectively.

The effective number of codon (ENC) values revealed a low codon usage bias in TTSuV, with mean values ± SD of 51.008 ± 1.28 for TTSuV1 and 45.534 ± 2.103 for TTSuVk2. Regarding the individual species, TTSuV1b (50.55 ± 1.13) displayed a higher codon usage bias compared to TTSuV1a (51.65 ± 0.92), while TTSuVk2a (45.16 ± 1.64) had a higher codon usage bias compared to TTSuVk2b (50.75 ± 0.36) (Figure 3). Overall, TTSuVk2a had the highest codon usage bias.

#### 2.3.4. Factors Shaping the Codon Usage of TTSuV

**Figure 4:**
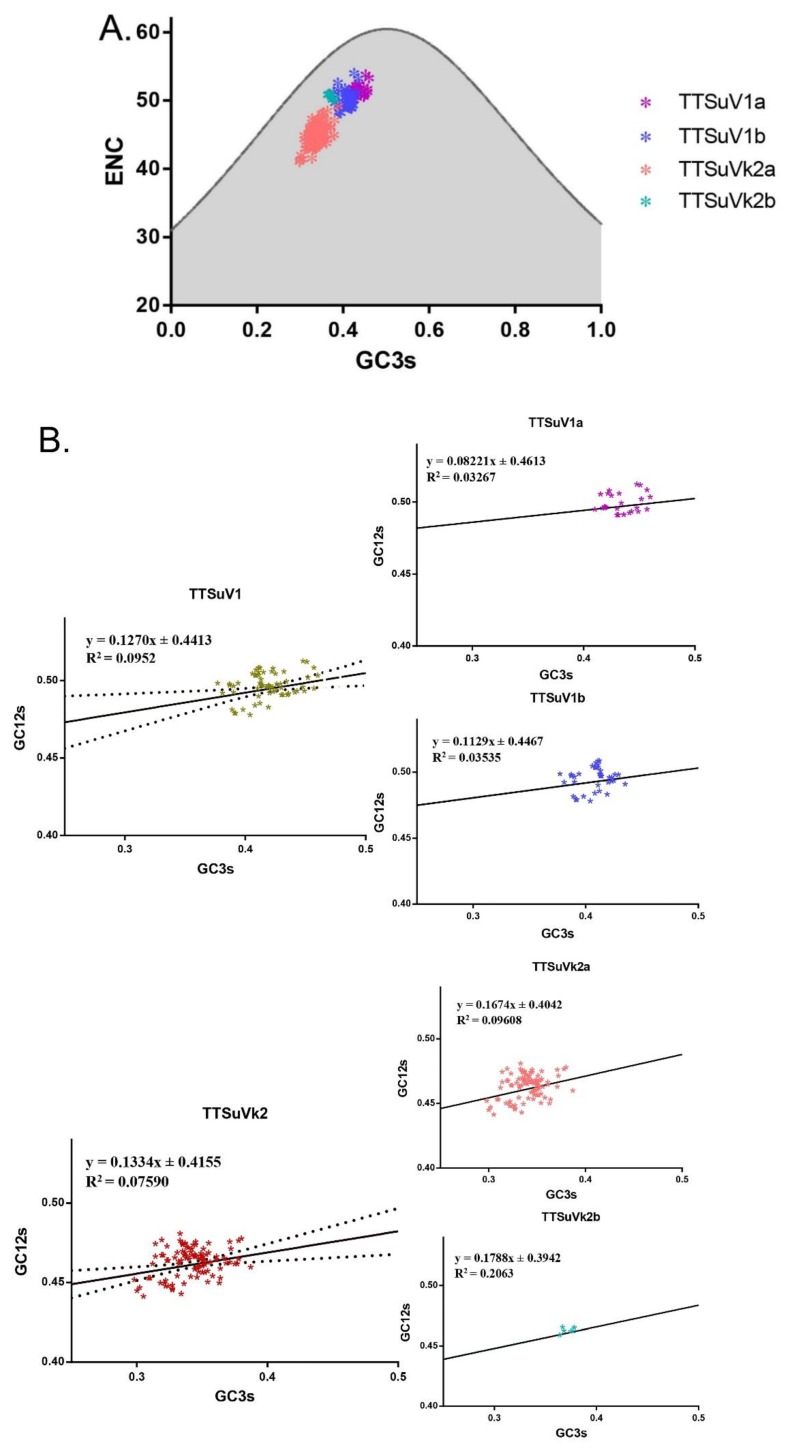
(**A**) ENC-plot representing GC3s plotted against the ENC of individual TTSuV species. (**B**) Neutrality analysis of individual TTSuV species, with GC12s plotted against GC3s. The dashed line indicates the 95% confidence interval. TTSuV1 is represented in green-brown, TTSuVk2 is represented in red, while TTSuV1a, TTSuV1b, TTSuVk2a, and TTSuVk2b are represented in purple, blue, pink, and green, respectively.

To further estimate factors influencing the low codon usage bias of TTSuV, an ENC-plot analysis and neutrality analysis were performed. ENC-plot analysis showed that all the TTSuV strains located under the standard curve, indicating that mutation pressure was not the sole force influencing codon usage. In particular, the analysis revealed general clustering among individual species (Figure 4A). 

Next, the level of contribution of mutation pressure and natural selection were investigated. GC12s and GC3s regression showed a significant difference (*p* < 0.05), with a slope of 0.1270 (R^2^ = 0.09522), indicating that mutation pressure accounts for 12.7%, while natural selection accounts for 87.3%. For TTSuVk2, we found a very significant difference (*p* < 0.001), with a slope of 0.1334 (R^2^ = 0.07590), indicating that mutation pressure accounts for 13.34% and 86.66% for natural selection. In terms of species-specific neutrality plots, the slopes were 0.08221 and 0.1129 for TTSuV1a and TTSuV1b, respectively, and 0.1674 and 0.1788 for TTSuVk2a and TTSuVk2b, respectively (Figure 4B). Overall, natural selection is the dominant factor shaping the evolution of different TTSuV species.

#### 2.3.5. Species-Specific Codon Adaptation and Deoptimization Pattern of TTSuV

**Figure 5:**
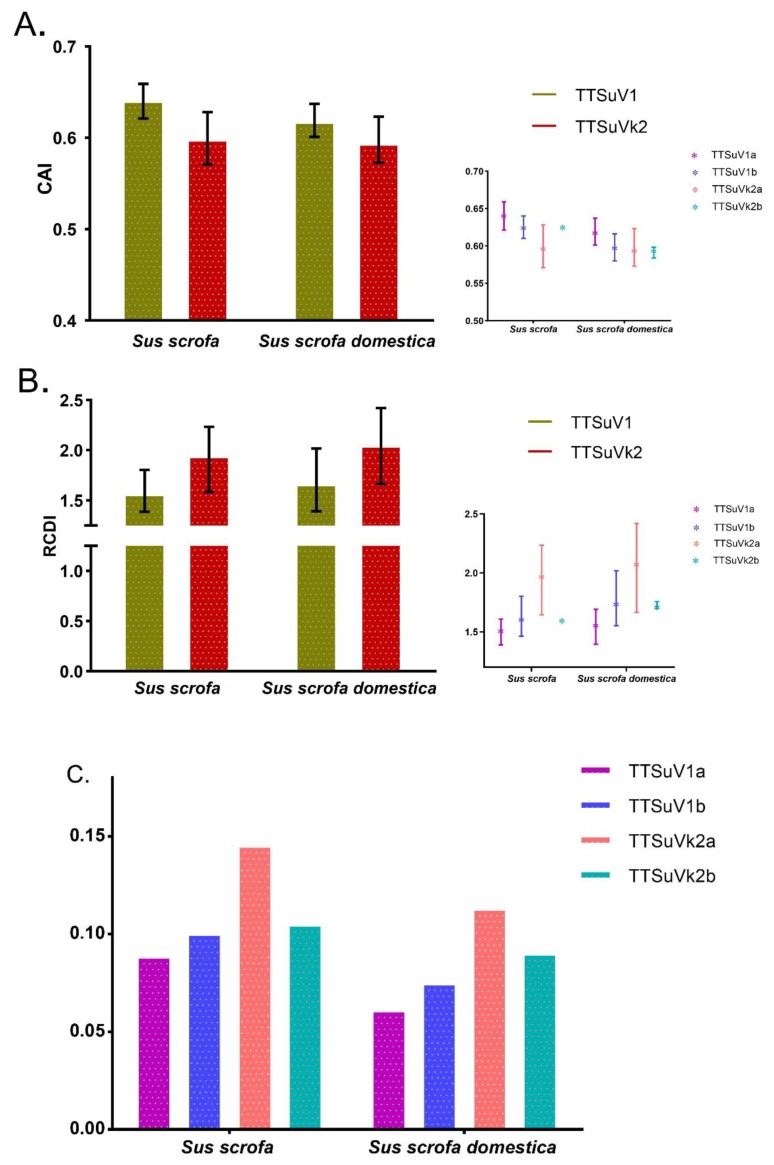
Codon adaptation index (CAI) (**A**), relative codon deoptimization index (RCDI) (**B**), and similarity index (SiD) (**C**) analysis of TTSuV. TTSuV1 is represented in green-brown and TTSuVk2 is represented in red, while TTSuV1a, TTSuV1b, TTSuVk2a, and TTSuVk2b are represented in purple, blue, pink and green, respectively.

Next, we explored the level of species adaptation and deoptimization. Firstly, we determined the codon adaptation index (CAI) to investigate the expression level of TTSuV in different hosts, including *Sus scrofa* and *Sus scrofa domestica*. TTSuV1 displayed a higher adaptation compared to TTSuVk2, with CAI values ranging from 0.601 to 0.659, while for TTSuVk2, values ranged from 0.571 to 0.628, regardless of hosts. We observed that TTSuV1a exhibited the highest CAI value, while TTSuVk2a exhibited the lowest. We found TTSuV to be more adapted to *Sus scrofa*, except for TTSuVk2a, whcih had similar values for *Sus scrofa* and *Sus scrofa domestica* (Figure 5A).

Regarding the codon deoptimization of TTSuV in respect to its hosts, TTSuVk2 displayed the highest deoptimization by relative codon deoptimization index (RCDI) for both *Sus scrofa* and *Sus scrofa domestica*, especially for TTSuVk2a. Furthermore, for TTSuV1, high RCDI values were observed in *Sus scrofa domestica* in comparison to *Sus scrofa* (values 1.665 and 1.569, respectively). For TTSuVk2, the values against *Sus scrofa* and *Sus scrofa domestica* were 1.947 and 2.052, respectively. In addition, regarding species-specific groups, TTSuVk2a displayed the highest deoptimization to *Sus scrofa domestica* (Figure 5B).

#### 2.3.6. Selection Pressure on TTSuV is Species-Specific

A similarity index (SiD) analysis was performed to detect the degree to which the hosts’, *Sus scrofa* and *Sus scrofa domestica,* codon usage pattern impacts the virus codon usage pattern. We found that, in comparison to *Sus scrofa domestica*, the role of *Sus scrofa* was more important in shaping the evolution of TTSuV, especially in TTSuVk2 (SiD values of *Sus scrofa domestica* on TTSuV1: 0.068 and in TTSuVk2: 0.132; SiD values of *Sus scrofa* on TTSuV1 and TTSuVk2: 0.094 and 0.149, respectively) (Figure 5C). In addition, the influence of *Sus scrofa* on the individual species was more important than *Sus scrofa domestica*, with a more significant role on TTSuVk2b.

### 2.4. TTSuV Dinucleotide Abundance

**Figure 6:**
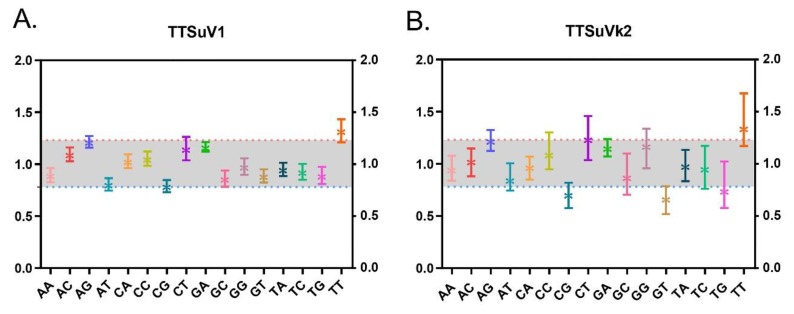
The 16 dinucleotide abundance of TTSuV1 (**A**) and TTSuVk2 (**B**). The pink dashed line represents the value 1.23, and the blue dashed line represents the value 0.78.

Dinucleotide composition revealed that there was preference on the usage of dinucleotides. None of the 16 dinucleotides were under-represented, with the lowest frequency for CpG (0.789 ± 0.058) and TpT being over-represented with a value of 1.32 ± 0.11 in TTSuV1. In contrast, a wider range of dinucleotides were identified in TTSuVk2. CpG, GpT, and TpG were under-represented with mean ± SD values of 0.706 ± 0.047, 0.668 ± 0.055, and 0.742 ± 0.091, respectively, and TpT and CpT were over-represented with mean ± SD values of 1.237 ± 0.047, and 1.344 ± 0.104, respectively (Figure 6).

## 3. Discussion

TTSuV in pig farms is considered to be ubiquitous worldwide. Although of uncertain pathogenicity, it still remains a threat to the porcine industry, based on previous studies showing its ability to worsen PCVAD [18,19]. Therefore, to avoid the potential risk to pig farms, a better understanding of the evolutionary changes of TTSuV is one of the important steps to develop preventive measures. Although previous studies performed codon usage analysis on TTSuV [20,21], no accurate comparison among different species nor correlation with adaptation were studied. Here, an extensive codon usage analysis based on the ORF1 gene was carried out for the first time to reveal the evolutionary changes and host-specific adaptation of the TTSuV species. 

Recombination analysis detected a potential intra-species recombination signal in the ORF1 gene, especially in TTSuVk2a, in agreement with previous reports [8]. Phylogenetic analysis of the ORF1 gene combined with the nucleotide divergence revealed an independent evolutionary process for the two genera. Importantly, PCA revealed similar independent distributions of individuals, except for several overlaps in TTSuV1a and 1b, which might indicate divergence from a common ancestor [22]. In terms of nucleotide composition, ATs were preferred over GCs, with nucleotide A being more abundant. Additionally, we revealed dinucleotide abundance differences. The CpG composition was the lowest and under-represented in TTSuV1, while for TTSuVk2, it was low but not the lowest among the 16 dinucleotides. CpG in relation to RSCU analysis showed that all the NCG and CGN synonymous codons were less than one, indicative of negative codon usage for both TTSuV1 and TTSuVk2. CpG deficiency is a reflection of unmethylated CpG. Unmethylated CpG act as signatures for the innate immune system [23]. Whether this is the case for TTSuVs remains to be invistigated.

Generally, multiple factors drive codon usage bias, with mutation pressure and natural selection accounting for the biggest effect in most species [24]. Overall, the codon usage of TTSuV was low for both TTSuV1 and TTSuVk2. However, it was higher in TTSuVk2 than in TTSuV1, especially in TTSuVk2a. Low codon usage bias might be beneficial for virus replication in hosts which have different codon usage patterns [25], such as the chicken anemia virus (CAV), belonging to the same family, the *Anelloviridae,* but previously classified as *Circoviridae* [26], which has an ENC value of 55 ± 0.93 based on the ORF1 gene [27]. The porcine circovirus 2 (PCV2) has an ENC value of 54.31 [28], while the PCV3 has an ENC value of 55.52 [29], which enables prevalence in swine. Therefore, low codon usage bias in TTSuV may facilitate replication. Additionally, we investigated the forces driving the low codon usage bias of TTSuV using ENC-plot analysis and neutrality analysis. We found that both mutation pressure and natural selection drive the evolution of TTSuV, especially natural selection, having a more dominant effect in TTSuV1 compared to TTSuVk2.

RSCU analysis revealed that A-ended synonymous codons were preferred in TTSuV, in line with the overall AT-rich composition. We also compared the RSCU pattern between TTSuV and hosts and found a mixed phenomenon with different magnitudes of coincidence and in-coincidences in species-specific groups with a high ratio of coincidence/antagonism in TTSuV1 to *Sus scrofa domestica*. Consistent codon usage patterns allow effective amino acid translation, while inconsistent codon usage patterns are beneficial for protein folding [30]. This phenomenon suggested host selection pressure and possible adaptation of TTSuV, especially TTSuV1 to *Sus scrofa domestica* [31].

CAI analysis reflects the gene expression level to host cells and thus the effect of natural selection on virus evolution [32]. We performed CAI, RCDI and SiD analysis against the reference hosts *Sus scrofa* and *Sus scrofa domestica*. CAI analysis revealed that TTSuV1 and TTSuVk2 experienced different evolution dynamics: TTSuV1 was more adapted to different host species, especially to *Sus scrofa*, while for TTSuVk2, the same CAI values were found, except for TTSuVk2b. In particular, TTSuV1 was more expressed in *Sus scrofa,* while the same expression level was identified in *Sus scrofa* and *Sus scrofa domestica* for TTSuVk2a. Low RCDI values indicated high expression or replication in hosts [31]. RCDI analysis of TTSuV revealed higher values in *Sus scrofa domestica*, compared to *Sus scrofa*, both for TTSuV1 and TTSuVk2, especially in *Sus scrofa domestica* for TTSuVk2a. Using SiD analysis, we found that *Sus scrofa* and *Sus scrofa domestica* imposed similar selection pressure on TTSuVk2, especially on TTSuVk2a. However, in TTSuV1, the role of *Sus scrofa* was more important than *Sus scrofa domestica*. Overall, the above results indicate the significant role of *Sus scrofa* in shaping the evolution of TTSuV, specially TTSuV1, and both *Sus scrofa* and *Sus scrofa domestica* in the TTSuVk2a, which might contribute to the high prevalence of TTSuVk2a worldwide. 

In conclusion, to explain the codon usage changes during the evolution of TTSuV, an extensive codon usage analysis was performed for the first time. We found that TTSuV1 and TTSuVk2 experienced different evolution dynamics. The low codon usage bias could benefit TTSuV host adaptation. The dominant role of natural selection might be one of the factors shaping the previously reported substitution rate [8]. In addition, CAI, RCDI, and SiD analysis uncovered the important role of *Sus scrofa* in the evolution of TTSuV, especially in TTSuV1a. Therefore, future pathogenicity studies should focus on TTSuV in *Sus scrofa*. 

## 4. Materials and Methods

### 4.1. Sequence Data

A total of 181 complete TTSuV ORF1 genes were downloaded from the National Center for Biotechnology Information (NCBI) GenBank (https://www.ncbi.nlm.nih.gov/genbank/) up to February 2019. Nucleotide sequences were aligned in amino acids and then translated to nucleotide in MEGA 7 (Arizona State, USA; Commonwealth of Pennsylvania, USA) [33].

### 4.2. Recombination and Phylogenetic Analysis

A recombination signal was detected with the Recombination Detection Program (RDP4) (Cape Town, South Africa) [34]. A total of seven methods, including RDP, GENECONV, Chimaera, MaxChi, BootScan, SiScan, and 3Seq were applied with default settings, except for the replication, which was 1000 and a cut off p value was 0.01. More than four methods were needed to identify recombination for the sequences to be considered recombinant and further confirmed by SimPlot (v3.5.1) (Maryland, USA) [35]. After removal of recombinant sequences, a maximum likelihood (ML) phylogenetic tree was inferred using RAxML (v8.2.10) (Heidelberg, Germany) [36], based on the general time-reversible (GTR)+I+Γ substitution model identified by ModelGenerator (Nottingham, UK) [37]. 

### 4.3. Codon Usage Analysis

#### 4.3.1. Sequence Composition

The characterization of the sequence composition of the TTSuV1 and TTSuV2 ORF1 gene coding sequences included: (i) Nucleotide composition (A%, T%, G%, C%) (calculated using BioEdit (California, USA)); (ii) nucleotide frequency at the first, second, and third position of synonymous codons (A3s%, T3s%, G3s%, C3s%); (iii) G+C at the first, second, third position of synonymous codons; (iv) overall GC and AT frequency. Points (ii), (iii), and (iv) were calculated using the software CodonW (Oxford, UK) (http://codonw.sourceforge.net/). Given that Met and Trp encode only for ATG and TGG, respectively, and that TGA, TAA, and TAG are stop codons, they were excluded from the analysis. 

#### 4.3.2. Relative Synonymous Codon Usage

The RSCU values of a synonymous codon refers to the relative probability of its observed frequency to its expected frequency, assuming that all codons for an amino acid are used equally, removing the effect of amino acid composition on the use of codons [38]. Equation (1):

(1) $$\mathrm{RSCU}=\frac{g_{ij}}{\sum_{j}^{n_{i}}g_{ij}}ni$$

where g_ij_ is the observed number of the i_th_ codon for the j_th_ amino acid, which has n_i_ kinds of alternative synonymous codons. A value of 1 is the boundary of the positive (>1) and negative (<1) codon usage. Values higher than 1.6 and less than 0.6, indicate over-represented and under-represented synonymous codons, respectively [39].

### 4.4. Principal Component Analysis

PCA is the representation of major tends of the codon usage pattern of a given coding sequence based on a multivariate statistical method. For the analysis, a 59-dimensional vector, which relates to the RSCU values of the 59 sense codons, represents each sequence [25]. Each axis value was calculated by CodonW.

### 4.5. Effective Number of Codons Analysis

ENC indicates the degree of codon usage bias, excluding gene length, as well as the occurrence of amino acids [25]. Here, it was calculated using the following formula (2):

(2) $$\mathrm{ENC}=2+\frac{9}{\bar{F_{2}}}+\frac{1}{\bar{F_{3}}}+\frac{5}{\bar{F_{4}}}+\frac{3}{\bar{F_{6}}}$$

where $F_{k}$ (k = 2,3,4,6) means the average *F_k_* in the k-fold degenerate amino acid family and was calculated with the formula (3):

(3) $$F_{k}=\frac{nS-1}{n-1}$$

where *n* means the summary of the codons for the corresponding amino acid. In addition, *S* was calculated as follows (4):

(4) $$S=\overset{k}{\underset{i=1}{\sum }}{\left(\frac{n_{i}}{n}\right)}^{2}$$

where $n_{i}$ means the total frequency of the *i*th codon for the corresponding amino acid. The ENC value ranged from 20 to 61, with the value 20 indicating extreme codon usage bias and 61 indicating no codon bias [40]. Thus, larger ENC values indicate lower codon usage bias. Here, an ENC value of less than 35 was considered as significant high codon usage bias [41]. 

Furthermore, to better understand factors shaping the codon usage bias, ENC-plot analysis with GC3s plotted against the ENC values was completed. Mutation pressure was considered to be the only factor constraining the codon usage when the observed value sat on the standard curve. Otherwise, other factors shaped codon usage [42]. The ENC values were calculated as follows (5):

(5) $$ENC^{expected}=2+s+\left(\frac{29}{s^{2}+\left(1-s^{2}\right)}\right)$$

where s means the percent of GC at the third position of synonymous codons (GC3s).

### 4.6. Neutrality Analysis

Neutrality analysis, the relationship between GC12s and GC3s, is commonly applied to distinguish the dominant role of mutation pressure and natural selection. The slope of the regression line when GC12s are plotted against GC3s indicates equilibrium in mutation-selection pressure. Thus, points distributed along the diagonal line indicate balance among the three codon positions, with no or little effect by selection pressure [43]. In general, the regression slope is the expression of the extent of neutrality.

#### Codon Adaptation Index and Relative Codon Deoptimization Index

CAI is considered to be a determination of the codon usage tendency of the virus to its corresponding hosts and displays the expression level of the respective coding sequence. With a range of 0–1, higher CAI values indicate higher preference, thus, more adaptation to hosts [44]. The CAI values were calculated using the CAIcal server (Tarragona, Spain) [45]. *Sus scrofa* and *Sus scrofa domestica* were used as reference hosts, with the relative synonymous codon usage of the two hosts retrieved from the Codon Usage Database (http://www.kazusa.or.jp/codon/). 

In contrast, RCDI is a measure of the tendency of the codon deoptimization of virus to its hosts. Here, it was calculated using the RCDI/eRCDI sever (Tarragona, Spain) [45]. A RCDI value more than 1, and closer to 1, means the virus is adapted to the host codon usage pattern [31].

### 4.7. Similarity Index

SiD is the measure that the host codon usage pattern has on shaping the virus codon usage pattern. It ranges from 0 to 1. A higher value indicates a more influencing role [46]. It was calculated using the following Formula (6) and (7):

(6) $$R\left(A,B\right)=\frac{\sum_{i=1}^{59}a_{i}*b_{i}}{\sqrt{\sum_{i=1}^{59}a_{i}{}^{2}*\sum_{i=1}^{59}b_{i}{}^{2}}}$$

(7) $$D\left(A,B\right)=\frac{1-R\left(A,B\right)}{2}$$

where $a_{i}$ is the virus RSCU value of an individual codon in the synonymous codon family and $b_{i}$ the same value of the reference host. R(A,B) is the exploration of codon usage similarity in the virus and relative host. D(A,B) indicates the influence of the host on the virus during evolution in terms of codon usage pattern [22].

### 4.8. Dinucleotide Abundance Analysis

The 16 dinucleotide abundance was calculated in the software DAMBE (Ottawa, Canada) [47], including the expected and observed frequencies. The comparison between the expected and observed value was performed using the following odds (8):

(8) $$P_{xy}=\frac{f_{xy}}{f_{y}f_{x}}$$

where $f_{xy}$ is the observed occurrence of dinucleotide XY and $f_{y}f_{x}$ is the expected occurrence of dinucleotide XY [48]. $P_{xy}$ more than 1.23 suggests over-represented dinucleotide abundance, while, $P_{xy}$ less than 0.78 suggests under-represented dinucleotide abundance.

## Figures and Tables

**Table 1:** RSCU values of TTSuV1 and TTSuVk2 and of the hosts, *Sus scrofa* and *Sus scrofa domestica*.

AA	Codon	TTSuV1	TTSuV2	*Sus scrofa*	*Sus scrofa domestica*
**F**	UUU	**1.1**	**1.2**	0.79	0.59
	UUC	0.9	0.8	**1.21**	**1.41**
**L**	UUA	0.93	1.73	0.32	0.3
	UUG	0.32	0.21	0.67	0.49
	CUU	0.79	0.57	1.35	1.41
	CUC	0.99	1.51	1.35	1.41
	CUA	**1.74**	**1.68**	0.33	0.28
	CUG	1.22	0.29	**2.68**	**2.92**
**I**	AUU	0.84	0.54	0.91	1.22
	AUC	0.6	0.49	**1.67**	**1.45**
	AUA	**1.56**	**1.97**	0.42	0.34
**V**	GUU	0.55	0.65	0.57	0.34
	GUC	0.6	0.6	1.07	1.45
	GUA	**1.81**	**2.24**	0.34	0.17
	GUG	1.04	0.51	**2.03**	**2.04**
**S**	UCU	0.96	0.71	0.99	1.1
	UCC	0.83	0.6	1.5	1
	UCA	1.28	1.51	0.73	**2.45**
	UCG	0.59	0.15	0.39	0.55
	AGU	**1.61**	1.02	0.77	0.25
	AGC	0.73	**2**	**1.62**	0.66
**P**	CCU	0.6	**1.57**	1.05	0.84
	CCC	0.86	0.42	**1.46**	**1.59**
	CCA	**1.9**	0.64	0.94	0.91
	CCG	0.65	0.71	0.56	0.66
**T**	ACU	0.69	**2.33**	0.83	0.77
	ACC	0.65	0.31	**1.68**	1.2
	ACA	**2.08**	1.14	0.92	**1.62**
	ACG	0.58	0.48	0.57	0.41
**A**	GCU	0.76	**2.16**	0.96	0.7
	GCC	1.07	0.22	**1.8**	1.03
	GCA	**1.6**	1.03	0.74	**1.92**
	GCG	0.57	0.98	0.5	0.36
**Y**	UAU	0.57	**1.14**	0.73	0.69
	UAC	**1.43**	0.86	**1.27**	**1.31**
**H**	CAU	0.56	0.72	0.7	0.6
	CAC	**1.44**	**1.28**	**1.3**	**1.4**
**Q**	CAA	0.81	**1.5**	0.44	0.29
	CAG	**1.19**	0.5	**1.56**	**1.71**
**N**	AAU	0.77	0.93	0.79	0.82
	AAC	**1.23**	**1.07**	**1.21**	**1.18**
**K**	AAA	**1.38**	**1.6**	0.76	0.91
	AAG	0.62	0.4	**1.24**	**1.09**
**D**	GAU	0.6	0.75	0.8	0.81
	GAC	**1.4**	**1.25**	**1.2**	**1.19**
**E**	GAA	**1.22**	**1.39**	0.72	**1.24**
	GAG	0.78	0.61	**1.28**	0.76
**C**	UGU	0.88	**1.3**	0.79	0.81
	UGC	**1.12**	0.7	**1.21**	**1.19**
**R**	CGU	0.48	0.15	0.44	0.4
	CGC	0.88	0.95	**1.31**	1.09
	CGA	0.82	0.64	0.6	0.27
	CGG	0.3	0.52	1.29	0.77
	AGA	**2.8**	**2.95**	1.12	**2.75**
	AGG	0.71	0.79	1.23	0.71
**G**	GGU	0.62	0.32	0.57	0.31
	GGC	0.55	0.5	**1.46**	0.84
	GGA	**2.21**	**2.74**	0.91	**1.78**
	GGG	0.61	0.44	1.05	1.07

Note: The most abundant synonymous codons encoding the same amino acid are represented in bold.

## Supplementary Materials

Supplementary materials can be found at https://www.mdpi.com/1422-0067/20/12/2881/s1.
