# Supplementary figures and images for: Genetic Analysis and Evolutionary Changes of the Torque teno sus Virus
Source: Int J Mol Sci. 2019 Jun 13;20(12):2881. doi: 10.3390/ijms20122881 (PMC6628323; doi:10.3390/ijms20122881)

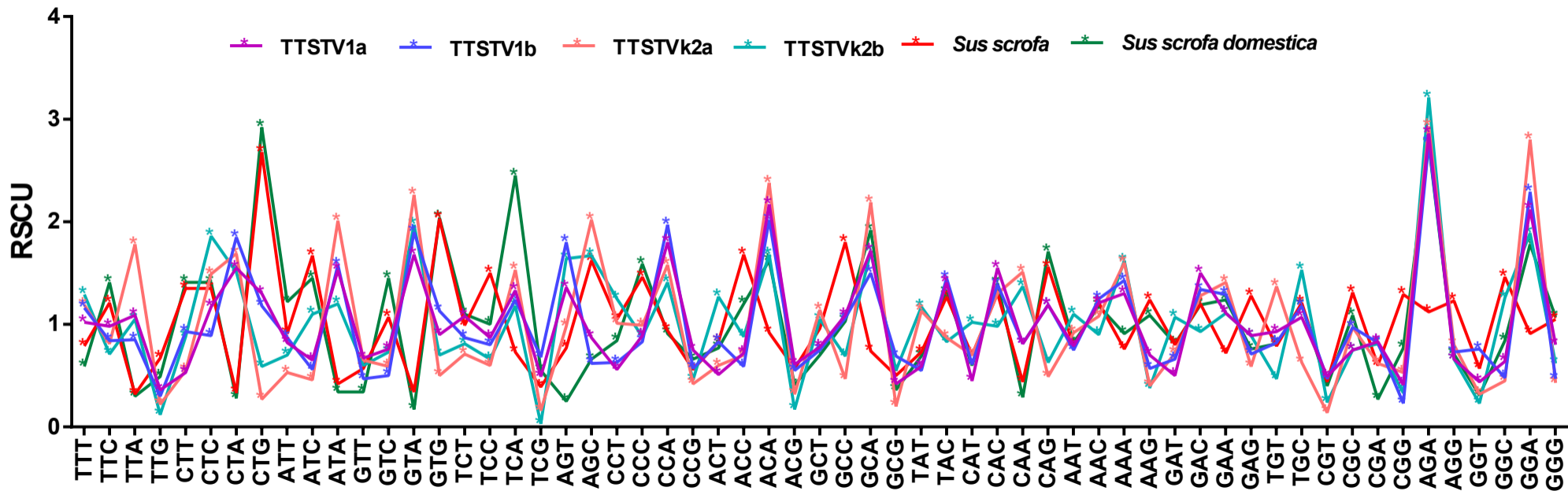

Supplement: Supplementary file 1 [file ijms-20-02881-s001.zip › Figure S1.pdf]
